# Long-Term Aerobic Exercise Enhances Hepatoprotection in MAFLD by Modulating Exosomal miR-324 via ROCK1

**DOI:** 10.3390/metabo14120692

**Published:** 2024-12-09

**Authors:** Yang Zhang, Qiangman Wei, Xue Geng, Guoliang Fang

**Affiliations:** Exercise Biological Research Center, China Institute of Sport Science, Beijing 100061, China; zhangyang@ciss.cn (Y.Z.); weiqiangman@ciss.cn (Q.W.); 2019112030@bsu.edu.cn (X.G.)

**Keywords:** non-alcoholic fatty liver disease (MAFLD), aerobic exercise, insulin resistance (IR), exosomes, miR-324, metabolic regulation

## Abstract

Background: Insulin resistance (IR) is central to the progression of non-alcoholic fatty liver disease (MAFLD). While aerobic exercise reduces hepatic fat and enhances insulin sensitivity, the specific mechanisms—particularly those involving exosomal pathways—are not fully elucidated. Method: Exosomes were isolated from 15 MAFLD patients’ plasma following the final session of a 12-week aerobic exercise intervention. Liver fat content was measured using MRI-PDFF, and metabolic parameters were assessed via OGTT, HOMA-IR, QUICKI, and VO_2_ max. Co-culture experiments evaluated the effects of exercise-derived exosomes on IR signaling pathways. miRNA microarray analysis identified miR-324, which was quantified in high-fat diet (HFD) mice with and without exercise and compared between athletes and sedentary controls. Functional assays assessed miR-324’s role in glucose and lipid metabolism, while luciferase reporter and Western blot assays confirmed ROCK1 as its direct target. Result: Aerobic exercise significantly reduced liver fat and improved insulin sensitivity in both MAFLD patients and HFD mice. Notably, exosomal miR-324 levels were lower in athletes than sedentary controls, indicating an inverse association with insulin sensitivity. Post-exercise, precursor and mature miR-324 increased in adipose tissue and decreased in muscle, suggesting its adipose origin and inverse regulation. Functional assays demonstrated that miR-324 modulates insulin resistance by targeting ROCK1. Conclusion: Exercise-induced exosomal miR-324 from adipose tissue targets ROCK1, revealing a novel mechanism by which aerobic exercise confers hepatoprotection against insulin resistance in MAFLD. These findings enhance our understanding of how exercise influences metabolic health and may inform future therapeutic strategies for managing MAFLD and related conditions.

## 1. Introduction

Metabolic associated fatty liver disease (MAFLD) remains a globally prevalent disease, with an incidence rate of approximately 25%, posing a significant threat to public health. Insulin resistance (IR) is recognized as the most critical pathological basis of the disease. However, current treatment strategies primarily rely on pharmacological interventions, such as fat absorption inhibitors, which have limitations in effectively addressing the underlying mechanisms [1].

Exercise training not only reduces the risk of fat accumulation and IR but also provides direct endogenous protective effects on metabolic health [2,3,4]. Aerobic Baduanjin, a traditional Chinese Qigong exercise combining rhythmic breathing with gentle movements, has been shown to improve cardiopulmonary function and metabolic health1. Due to its ease of learning and suitability for all age groups, Baduanjin has been widely applied in exercise intervention studies. However, the precise mechanisms underlying exercise-induced energy mobilization and hepatic IR protection remain unclear. Current research suggests that these effects may be mediated through mechanisms such as cellular metabolic regulation, functional modulation, and muscle-derived factors with both metabolic and anti-inflammatory effects [5,6,7,8]. Muscle, adipose tissue, and the liver, as the major insulin-responsive organs, secrete exercise-induced factors that coordinate the systemic effects of exercise [9,10,11]. In recent years, research on these exercise-induced factors has gained considerable attention, but many questions regarding their nature and mechanisms of action remain unanswered.

Exosomes, a specialized subset of extracellular vesicles (EVs), are secreted by all types of cells and are present in various bodily fluids such as blood, urine, cerebrospinal fluid, synovial fluid, and saliva [12]. Exosomes consist of two major components: membrane proteins essential for their structural integrity and vesicle formation, and cargo that includes organ- and tissue-specific molecules [13]. These nanovesicles, typically 30 to 150 nm in size, carry and deliver a wide range of bioactive molecules, including proteins, lipids, RNA, and DNA. Given that circulating exosomes reflect the body’s physiological and pathological states and exist in significant quantities (10^10^ per milliliter), investigating the functional effects of exercise-induced circulating exosomes is of great significance [14,15], particularly their role in long-term exercise-induced hepatic IR protection [16].

D’Souza et al. demonstrated that a single session of high-intensity interval exercise significantly upregulated the expression of nine exomiRs, including miR-126-3p, miR-23a-3p, and miR-208a-3p, in plasma-derived exosomes [17]. This finding suggests that exercise modulates miRNA sorting during exosome biogenesis, selectively promoting the expression of specific exomiRs. Additionally, long-term aerobic exercise (4-week swim exercise) has been shown to upregulate cardioprotective exomiR miR-342-5p in cardiomyocyte-derived exosomes in diabetic db/db mice, inhibiting apoptotic signaling pathways (Caspase 9 and Jnk2) and enhancing survival signaling (p-Akt) [18].

Recent studies have revealed that adipose tissue releases adipose-derived exosomes (AT-exosomes), which contain gene regulatory elements targeting the liver, heart, and other organs [19,20,21]. Obesity, characterized by adipose tissue hypertrophy, alters the distribution of miRNAs in plasma exosomes, thereby affecting glucose and lipid metabolism homeostasis [22]. Excessive adipose tissue proliferation disrupts exosomal miRNA profiles, impairing the homeostasis of target organs and glucose-lipid metabolism [23]. Therefore, this study aims to investigate how long-term aerobic Baduanjin exercise modulates the cargo of adipose-derived circulating exosomes, particularly miR-324-5p, and its impact on hepatic insulin resistance (IR). By addressing the underlying molecular mechanisms, this study seeks to fill critical gaps in our understanding of the systemic effects of exercise and provide novel insights into the therapeutic potential of exercise-induced exosomal signaling.

## 2. Materials and Methods

### 2.1. Subjects

This study was conducted in accordance with the principles of the Declaration of Helsinki and received approval from the Ethics Committee of the National Sports Administration’s Institute of Sports Science. Informed consent was obtained from all participants. The study included two distinct populations:

(1) Fifteen postmenopausal women aged 55–65 years with metabolic-associated fatty liver disease (MAFLD) were recruited from the community. We selected women in this age range because they are typically postmenopausal with significantly reduced estrogen levels (FSH > 40 IU/L and estradiol [E2] < 20 pg/mL), affecting adipose tissue function and metabolism—key factors in our research. This age range minimizes hormonal fluctuations and ensures participants can safely tolerate the exercise intervention without increased risk of comorbidities.

(2) Twenty young women aged 19–25 years, consisting of ten elite athletes and ten sedentary controls. The elite athletes were national first-class athletes in track and field events, training intensively (approximately 20–25 h per week) for over one year. The sedentary controls had not engaged in regular exercise for at least two months.

### 2.2. Dietary Habits

All participants maintained their habitual diet throughout the study to reflect real-life conditions. Dietary recall questionnaires indicated that the habitual diet of the postmenopausal group primarily consisted of carbohydrates (e.g., rice, noodles, and bread), moderate protein intake (e.g., eggs, meat, tofu, and legumes), and higher fat intake (e.g., cooking oils and animal-based products).

### 2.3. Animals

Twelve-week-old male C57BL/6J mice were sourced from Beijing Vital River Laboratory Animal Technology Co., Ltd., (Beijing, China) and were selected for their susceptibility to diet-induced obesity and metabolic diseases. The high-fat diet (HFD), supplied by Beijing HFK Bioscience Co., Ltd. (Beijing, China), consisted of 60% kcal from fat, 20% from carbohydrates, and 20% from protein. Mice were fed the HFD continuously for 8 weeks to establish the metabolic associated fatty liver disease (MAFLD) model. Three mice from each group (control and MAFLD model) were randomly selected for validation. Following successful model establishment, all mice underwent a 6-week exercise intervention. All surgical procedures and animal care adhered to the “Guidelines for the Care and Use of Laboratory Animals” published by the National Institutes of Health (NIH Publication No. 85-23, revised 1996).

### 2.4. Training Protocol

Subjects: the training regimen for the fifteen subjects in the exercise group, all MAFLD patients, involved progressive intensity adjustments. Heart rate was monitored using Huawei smartwatches, starting at 60% of maximal oxygen uptake (VO_2_ max) and gradually increasing to 75%. Exercise duration was progressively increased from 45 min to 60 min per session, and frequency escalated from three sessions per week to five. All training sessions were conducted in the morning.

Animals: Based on previous studies, a 6-week exercise intervention in rodents can induce significant physiological and metabolic changes. Due to differences in lifespan and metabolism, this is equivalent to long-term exercise in humans. Therefore, this intervention duration effectively simulates the chronic exercise benefits relevant to human health. The exercise intervention was preceded by a 3-day treadmill adaptation period, during which speed and duration were gradually increased to acclimate the mice, and unfit subjects were removed. Following adaptation, mice underwent a 6-week formal treadmill exercise regimen at a 0° incline, with each session lasting 50 min. Each session was structured as follows: 10 min at 8 m/min, 30 min at 12 m/min, and 10 min at 13 m/min. Training was conducted 5 days per week.

### 2.5. MRIPDFF (Magnetic Resonance Imaging Proton Density Fat Fraction) Assessment

MRIPDFF is utilized for the quantitative evaluation of fat content in tissues, particularly for assessing the degree of hepatic fat infiltration. Initially, multi-slice images of the livers of MAFLD subjects were acquired using MRI equipment from the Zhongshan Hospital. Subsequently, specialized software was employed to analyze fat and water signals in these images. The proton density fat fraction (PDFF) is calculated by measuring the ratio of fat signal to the total signal (the sum of fat and water signals), with PDFF values typically expressed as percentages.

### 2.6. Positron Emission Tomography (PET) Combined with the Fat Metabolism Tracer *[^18^F]*-FTHA

This method was utilized to assess fat distribution and metabolic activity in mice. The experiment involved four groups of mice: a normal diet group (Chow), a normal diet plus exercise group (Chow + EXE), a high-fat diet group (HFD), and a high-fat diet plus exercise group (HFD + EXE). Two hours prior to the conclusion of the experiment, mice were administered an intravenous injection of [^18^F]-FTHA, followed by imaging analysis of systemic fat distribution using a PET scanner. After scanning, images were processed with dedicated software to quantitatively evaluate fatty acid metabolism across the groups, with a particular focus on the impact of exercise intervention on fat metabolism in FFD mice.

### 2.7. Maximal Oxygen Uptake

Maximal oxygen uptake during brisk walking was calculated using the 2-km walking index, a common cardiopulmonary function assessment method in Finland and other European countries. Participants were instructed to complete a 2 km course as quickly as possible on flat ground. Upon completion, the time taken and heart rate were recorded to compute the cardiopulmonary function index using the formula:

VO_2_ max (mL/min/kg) = 116.2 − 2.98 × walking time (s) − 0.11 × heart rate − 0.14 × age − 0.39 × BMI

The results are influenced by factors such as age, gender, height, and weight, allowing for the development of personalized exercise protocols based on the data obtained from the 2 km walking test.

### 2.8. Insulin Resistance Index (HOMA-IR) and Quantitative Insulin Sensitivity Check Index (QUICKI) *[24,25,26]*

In this study, HOMA-IR and QUICKI were employed to assess insulin sensitivity and resistance. HOMA-IR is calculated using the following formula:

HOMA-IR = Fasting insulin (mU/L) × Fasting glucose (mmol/L)/22.5

QUICKI is calculated using the following formula:

QUICKI = 1/log(Fasting insulin mU/L + log(Fasting glucose mg/dL)

Fasting blood glucose and insulin levels were measured using the standard glucose oxidase method and enzyme-linked immunosorbent assay (ELISA), respectively. All procedures were carried out according to the manufacturer’s protocols provided with the assay kits. The HOMA-IR was employed to assess insulin resistance, while QUICKI was used to evaluate insulin sensitivity.

### 2.9. Exosome Isolation from Plasma

Blood samples were collected from patients and participants 24 h after the final training session. For exosome isolation using sucrose density gradient centrifugation, all blood samples were centrifuged at 1600× *g* for 20 min at 4 °C to obtain plasma, followed by centrifugation at 10,000× *g* for 30 min at 4 °C to remove cells and platelets. The plasma was then centrifuged twice at 100,000× *g* for 60 min at 4 °C using an SW-41 rotor. Plasma exosomes were isolated using the ExoQuick Plasma Preparation and Exosome Precipitation Kit (System Biosciences, Palo Alto, CA, USA), following the manufacturer’s instructions. The isolated exosomes were resuspended in phosphate-buffered saline (PBS) for further experiments.

### 2.10. Construction of the Insulin Resistance Cell Model

Based on previous work, HepG2 cells were treated with 33.3 mmol/L glucose or a mixture of 500 µM palmitic acid, oleic acid, and insulin for 48 h. The cells were divided into a normal control group and a high-glucose treatment group.

### 2.11. miRNA Library Construction and Sequencing

miRNA library preparation and sequencing were conducted by a commercial service provider (Ribobio, Guangzhou, China). Briefly, total RNA was extracted from exosomes purified from 2 mL of plasma. Adaptors were added to both the 3′ and 5′ ends of the RNA, followed by reverse transcription polymerase chain reaction (RT-PCR) amplification. PCR products, originating from 18–30 nucleotide RNA molecules, were purified by electrophoresis and sequenced using the Illumina HiSeq 2500 platform.

### 2.12. Primary Hepatocyte Isolation and Culture

Mice were first weighed and anesthetized via intraperitoneal injection. After fixing the mice, the abdomen was opened to locate the portal vein, through which D-Hanks solution pre-warmed to 37 °C was perfused until the liver turned pale. Collagenase solution was then slowly injected through the portal vein for digestion (3–5 min). Once digestion was complete, the liver was excised, and the gallbladder was removed. The liver was cut into small pieces, and the tissue was passed through a metal sieve to obtain a hepatocyte suspension. The suspension was centrifuged (4 °C, 500 rpm, 5 min), the supernatant discarded, and the process repeated. The cells were then resuspended in medium. Hepatocytes were seeded in six-well plates or culture flasks, with the medium being changed 48 h after seeding and subsequently every two days.

### 2.13. Glycogen Assay

The glycogen assay was performed using a glycogen detection kit. Cultured cells were collected and washed with PBS 2–3 times. The cells were then lysed with lysis buffer, and the resulting suspension was boiled in water for 20 min to hydrolyze glycogen, followed by cooling and neutralization. The glycogen solution was diluted to 1% and mixed with the detection reagent. After heating in a boiling water bath for 5 min, the reaction was cooled, and the optical density (OD) was measured at 620 nm. Glycogen content was calculated based on a standard curve or formula.

### 2.14. Glucose Detection

Glucose concentration was measured using enzyme-linked immunosorbent assay (ELISA). Cells were seeded in 96-well plates, and after preparing the standards and samples, reagents were mixed according to the protocol. The reaction mixture was heated in a boiling water bath for 8 min, followed by cooling for 4 min. Subsequently, 200 µL of the reaction solution was added to each well of the 96-well plate, and absorbance was measured at 620–650 nm, with a peak wavelength at 630 nm. The procedure for cuvette detection was similar, where absorbance was read after adding the mixture. Glucose concentration in the samples was calculated based on a standard curve.

### 2.15. Pathway Analysis of miR-324 and ROCK1

To investigate the potential regulatory relationship and molecular mechanisms between miR-324 and ROCK1, pathway analysis was conducted using [specific tools, e.g., KEGG (Kyoto Encyclopedia of Genes and Genomes), GO (Gene Ontology), or other databases/software]. The predicted targets of miR-324 were identified using [e.g., TargetScan, miRDB], and functional enrichment analysis was performed to identify pathways involving ROCK1. Additionally, experimental validation was conducted via [e.g., luciferase reporter assay, qPCR, Western blot] to confirm the interaction between miR-324 and ROCK1.

### 2.16. Statistical Analysis

All data are presented as the mean ± SEM from n independent experiments. Statistical comparisons between two groups were performed using an unpaired, two-tailed Student’s *t*-test, while comparisons among three or more groups were analyzed using one-way analysis of variance (ANOVA), followed by Bonferroni correction when necessary. The levels of significance are indicated in the figures and text as follows: *p* < 0.05 (*), *p* < 0.01 (**), and *p* < 0.001 (***). All statistical analyses were conducted using GraphPad Prism version 5.0, with *p* < 0.05 considered statistically significant.

## 3. Results

### 3.1. Protective Effects of 12 Weeks of Aerobic Baduanjin Training on MAFLD Patients

**Figure 1:**
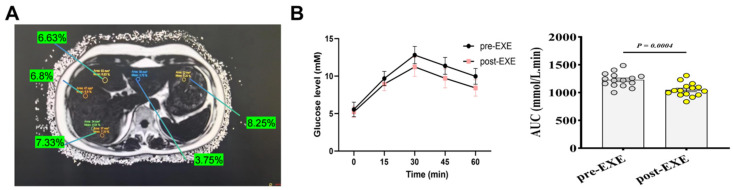
MRI-PDFF effects and OGTT results of 12-week aerobic exercise on MAFLD patients. (**A**) MRI-PDFF imaging showing reduction in liver fat content after 12 weeks of aerobic exercise in MAFLD patients. (**B**) OGTT results indicating improved glucose tolerance in MAFLD patients post-training.

After 12 weeks of aerobic Baduanjin training, MAFLD patients showed significant reductions in liver fat content, as detected by MRI-PDFF (Table 1). Visceral fat significantly decreased from 37.83 ± 1.74 to 32.73 ± 1.88 (*p* < 0.05). VO_2_ max significantly increased from 22.7 ± 1.96 to 26.03 ± 2.47 mL·kg^−1^·min^−1^ (*p* < 0.01). Fasting insulin levels significantly decreased from 20.8 ± 2.93 to 17.53 ± 2.23 μU/mL (*p* < 0.05), and the HOMA-IR index was significantly reduced from 5.17 ± 1.59 to 4.07 ± 1.09 (*p* < 0.05). QUICKI index significantly increased (*p* < 0.05), and HbA1c levels decreased significantly from 6.61 ± 0.49% to 6.17 ± 0.33% (*p* < 0.05). Fasting C-peptide levels were also significantly reduced (*p* < 0.05). Additionally, 24 h after the final session, oral glucose tolerance test (OGTT) results indicated improved glucose tolerance, with a significant reduction in the area under the curve (AUC) from 1200 ± 150 to 1020 ± 130 mmol·L^−1^·min (*p* < 0.05), indicating reduced glucose intolerance. Although there were trends of reduction in body weight, BMI, systolic blood pressure, and diastolic blood pressure, these changes were not statistically significant. Similarly, blood markers such as triglycerides (TG), total cholesterol (TCHOL), HDL cholesterol (HDL-CH), fasting glucose (GLU), aspartate aminotransferase (AST), and alanine aminotransferase (ALT) showed trends of reduction but without statistical significance (Table 1). MRI-PDFF imaging of liver fat and OGTT results (Figure 1A,B) suggest that 12 weeks of aerobic Baduanjin exercise confers protective effects against insulin resistance (IR)-induced liver damage.

### 3.2. Exercise Intervention Significantly Improves Glucose and Lipid Metabolism and Insulin Resistance in HFD-Fed Mice

**Figure 2:**
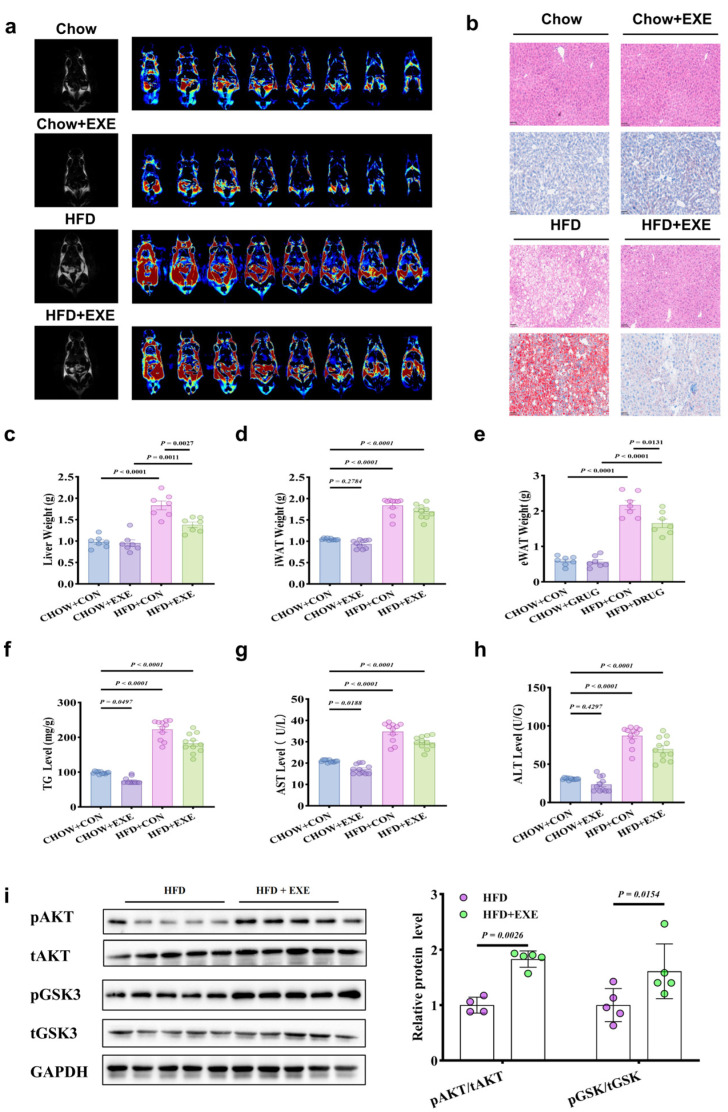
(**a**) pet imaging with [^18^F]-ftha showing reduced fat accumulation in the HFD + exe group. (**b**) liver histology (H&E, oil red o, and Masson’s staining) showing decreased lipid deposition and fibrosis in the HFD + exe group. (**c**–**e**) weights of liver (**c**), inguinal fat (iwat) (**d**), and epididymal fat (ewat) (**e**) reduced in the HFD + exe group. (**f**–**h**) improved liver triglycerides (tg) (**f**), ast (**g**), and alt (**h**) in the HFD + exe group. (**i**) increased liver insulin signaling (pAKT and pgsk3) in the HFD + exe group.

In the Chow group, body weight and fasting glucose levels did not show significant changes. However, in the HFD group, body weight significantly decreased post-exercise, and fasting glucose levels fluctuated (Supplementary Figure S1a,b). At week 20, OGTT analysis revealed significant glucose intolerance in the HFD group. After four weeks of exercise starting at week 22, glucose tolerance and insulin sensitivity in the HFD group improved significantly, with marked changes in AUC (Supplementary Figure S1c). HFD led to increased insulin resistance and enhanced gluconeogenesis in mice. In the HFD group, liver weight increased, and significant fat accumulation was observed (Figure 2a). However, after exercise intervention (HFD + EXE), liver volume significantly decreased, and fat accumulation was substantially reduced. Compared with the normal diet (Chow) and HFD exercise intervention groups (Chow + EXE), exercise effectively alleviated HFD-induced liver fat deposition (Figure 2b), confirming the protective effects of exercise on liver steatosis. Compared with controls, the HFD group showed significantly increased body weight, liver weight, eWAT, and iWAT weights (*p* < 0.0001) (Figure 2c–e). Following exercise intervention (HFD + EXE), these parameters significantly decreased (*p* < 0.0001). Moreover, exercise intervention significantly reduced serum triglyceride (TG), ALT, and AST levels in the HFD group (*p* < 0.05) (Figure 2f–h), indicating that exercise mitigates HFD-induced liver fat accumulation and liver dysfunction. Western blot analysis further revealed that the ratios of phosphorylated AKT (pAKT) to total AKT and phosphorylated GSK3 (pGSK3) to total GSK3 were significantly higher in the HFD + EXE group than in the HFD group (*p* < 0.05), suggesting that exercise intervention significantly enhanced insulin signaling activity (Figure 2i). Overall, HFD had a profound impact on metabolic health, while exercise effectively improved HFD-induced metabolic dysregulation.

After six weeks of aerobic exercise, the HFD + EXE group exhibited a significant reduction in body weight (*p* < 0.01), while the HFD group continued to gain weight (Supplementary Figure S1a). Fasting glucose levels in the HFD + EXE group were significantly lower than those in the HFD group (*p* < 0.01) (Supplementary Figure S1b). At week 20, OGTT results showed that glucose levels in the HFD + EXE group were significantly lower than in the HFD group, with a significant reduction in AUC (*p* < 0.01) (Supplementary Figure S1c). Insulin tolerance also significantly improved, as reflected in the significantly lower AUC in the insulin tolerance test (ITT) (*p* < 0.05) (Supplementary Figure S1d). Additionally, exercise intervention effectively suppressed pyruvate-induced gluconeogenesis (Supplementary Figure S1e). Exercise significantly reduced visceral fat volume (*p* < 0.001) and body fat percentage (*p* < 0.001) (Supplementary Figure S1f).

### 3.3. Identification of Plasma-Derived Exosomes and In Vitro Functional Validation

**Figure 3:**
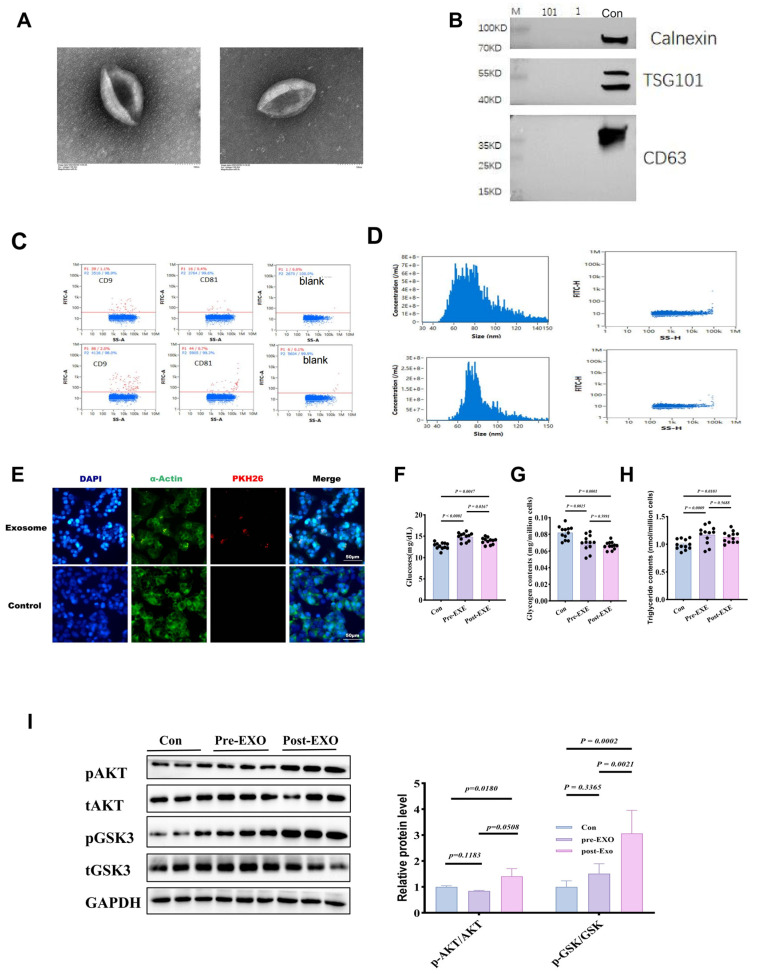
Characterization and functional validation of plasma-derived exosomes in exercise-conditioned individuals. (**A**–**D**) Exosomal morphology, surface markers, and concentration, confirming the purity and consistency of exosome isolation. (**E**–**H**) Uptake of exosomes by HepG2 cells and improvements in glucose uptake, glycogen, and triglyceride levels, indicating potential insulin resistance mitigation. (**I**) Western blot showing enhanced insulin signaling in cells treated with exercise-derived exosomes.

Twenty-four hours after the final training session, exosomes were isolated from patients’ plasma (Exe-exo) using a standard protocol of gradient and ultracentrifugation. Electron microscopy revealed typical round particles with diameters of 30–100 nm (Figure 3A). Western blot confirmed the presence of exosomal markers (TSG101 and CD81), with no significant differences in exosome quantity between Sed-exo and Exe-exo (Figure 3B). Nanoparticle tracking analysis similarly showed no significant differences in size distribution or plasma concentration between Sed-exo and Exe-exo (83.8 nm vs. 85.5 nm, 6.64 × 10^10^ vs. 5.03 × 10^10^, respectively) (Figure 3C,D). PKH26-labeled exosomes were incubated with HepG2 cells, and confocal imaging showed internalization of exosomes after 6 h of incubation in primary hepatocytes (Figure 3E). Notably, pre-incubation with Exe-exo (but not Sed-exo) for 48 h attenuated high-fat-induced insulin resistance in HepG2 cells, as evidenced by improved glucose, glycogen content, and triglyceride levels in the post-EXE exosome group compared to Pre-EXE exosomes (Figure 3F–H). Western blot analysis showed reduced P-AKT/GSK expression (Figure 3F), indicating that exercise-derived plasma exosomes protect against high-fat-induced dysregulation of hepatic glucose and lipid metabolism.

### 3.4. MiR-324 Is a Key Exogenous Factor in Protecting Against Insulin Resistance (IR) and Regulating Lipid and Glucose Metabolism

**Figure 4:**
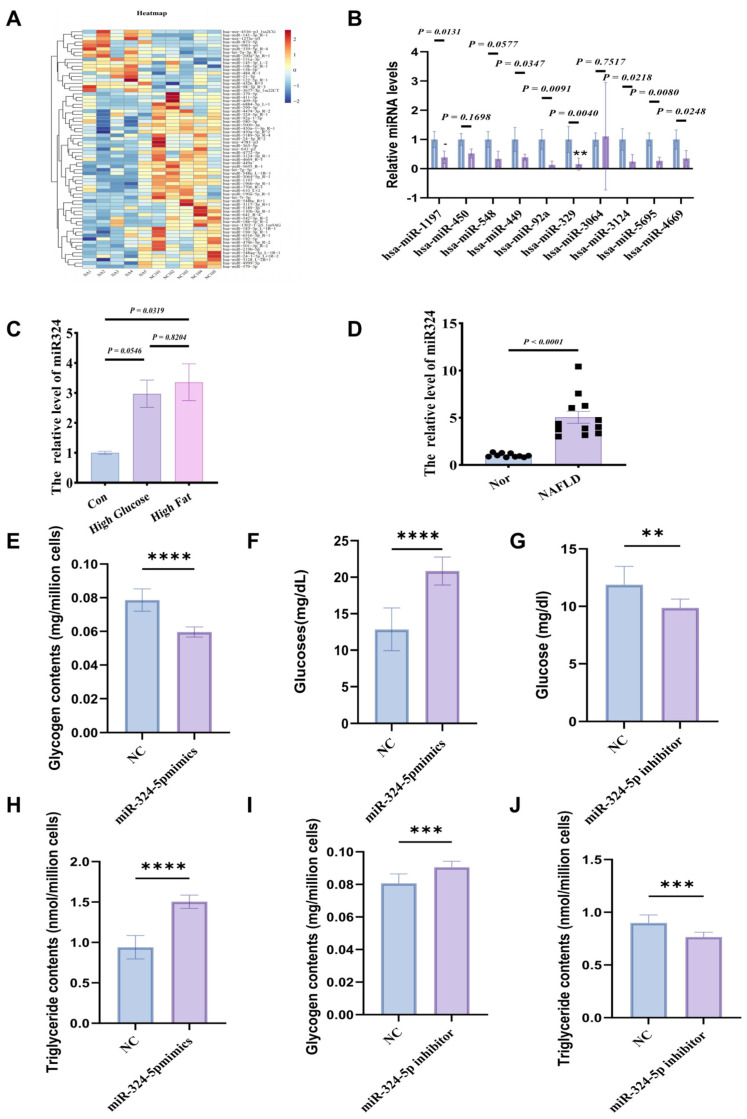
MiR-324-5p as a key exosomal miRNA regulating glucose and lipid metabolism. (**A**,**B**) Heatmap and relative expression of miRNAs, identifying miR-324-5p as significantly upregulated in exercise-derived exosomes. (**C**,**D**) Elevated miR-324-5p in high-glucose/high-fat-treated cells and MAFLD patients. (**E**–**J**) Functional effects of miR-324-5p overexpression and inhibition on glycogen and triglyceride content in HepG2 cells, demonstrating its role in metabolism regulation. *p* < 0.01 (**), *p* < 0.001 (***), and *p* < 0.0001 (****). All statistical analyses were conducted using GraphPad Prism version 5.0, with *p* < 0.05 considered statistically significant.

To explore the changes in miRNA content within exercise-induced exosomes and the potential for IR protection, ExomiRNA profiling was conducted using Illumina HiSeq 2500 (n = 5), comparing the differences between Exe-exo and Sed-exo. A total of 47 differentially expressed miRNAs were detected (absolute logFC > 2.0; *p* < 0.05; Figure 4A), with the top 10 upregulated miRNAs after exercise. Based on bioinformatics analysis, both GO and KEGG analyses revealed that miR-324 is closely associated with metabolic functions, making it an intriguing candidate. PCR results showed that miR-324 exhibited the greatest differential expression between the HFD + EXE-EXO and Sed-EXO groups. Furthermore, in high-glucose and high-fat-induced IR HepG2 cells, the expression level of miR-324-5p increased, aligning with the sequencing data (Figure 4C).

Next, we evaluated the functional role of miR-324-5p in regulating glucose and lipid metabolism through phenotypic experiments. The results showed that in insulin resistance models overexpressing miR-324-5p, glycogen content and glucose levels significantly increased (Figure 4D–F). Additionally, triglyceride levels were elevated. Conversely, inhibiting miR-324-5p expression led to a significant reduction in glycogen and triglyceride content (Figure 4E–G). These phenotypic data confirm the positive regulatory role of miR-324-5p in glucose and lipid metabolism.

### 3.5. MiR-324 Targets ROCK1 and Modulates Insulin Signaling Pathway

**Figure 5:**
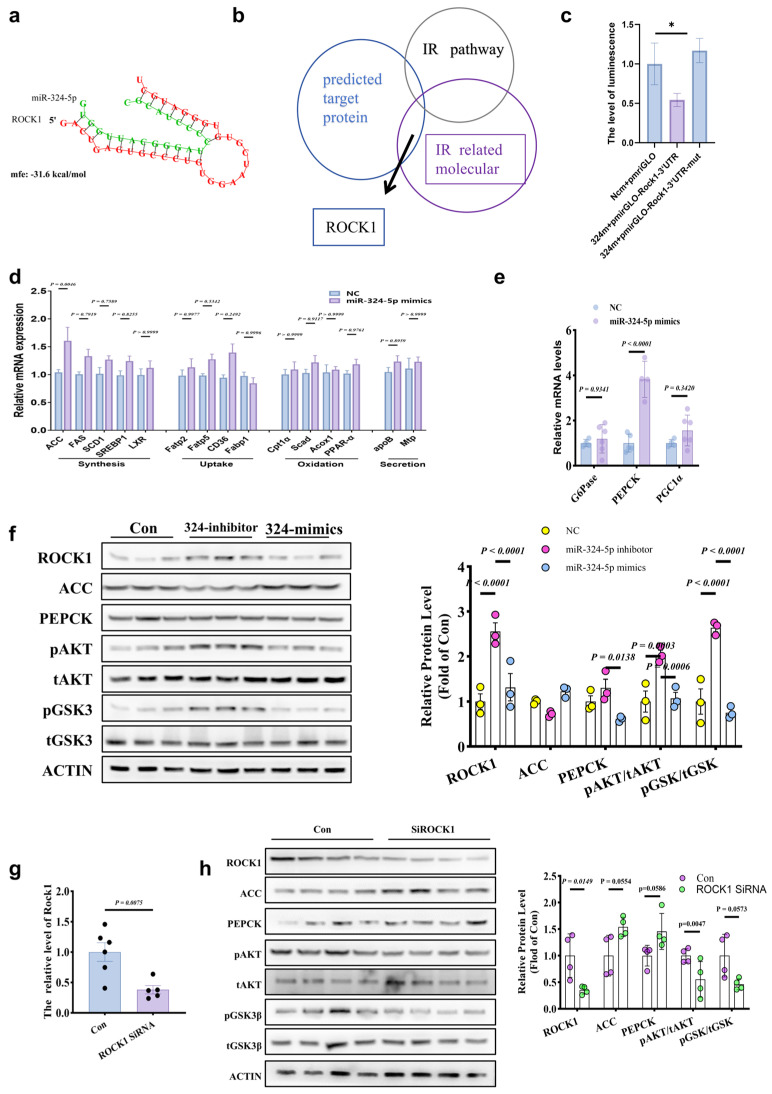
MiR-324-5p targets ROCK1 to modulate insulin signaling pathways. (**a**–**c**) miR-324-5p binding to ROCK1 3′UTR confirmed by luciferase assay, identifying ROCK1 as a direct target. (**d**,**e**) Upregulation of ACC and PEPCK with miR-324-5p overexpression. (**f**–**h**) Western blot analysis showing ROCK1 downregulation, enhanced insulin signaling, and similar effects in ROCK1 siRNA-treated cells. *p* < 0.05 (*). All statistical analyses were conducted using GraphPad Prism version 5.0, with *p* < 0.05 considered statistically significant.

Using secondary structure prediction tools miRanda and TargetScan, coupled with gene functional classification analysis in DAVID, we found that miR-324-5p is highly complementary to the 3′UTR region of ROCK1, with a binding energy of –31.6 kcal/mol, suggesting strong binding stability (Figure 5a,b). To further validate whether miR-324-5p regulates insulin signaling via ROCK1 targeting, we conducted a luciferase reporter assay. HepG2 cells were transfected with luciferase plasmids containing either the ROCK1 3′UTR or its mutated version, along with overexpressed miR-324-5p. Results showed that the luciferase activity of the ROCK1 3′UTR construct was significantly reduced, while the mutant construct exhibited no notable change (Figure 5c), confirming that ROCK1 is a direct target of miR-324-5p. Regarding glucose and lipid metabolism, qRT-PCR analysis demonstrated that overexpression of miR-324-5p significantly upregulated ACC and PEPCK expression levels, though changes in other lipid-related (synthesis, oxidation, and secretion genes) and glucose metabolism genes (G6P, PGC1) were observed but not statistically significant (Figure 5d,e). Western blot analysis further revealed that miR-324-5p overexpression markedly downregulated ROCK1 protein levels, with concomitant increases in ACC and PEPCK expression and alterations in key molecules of the insulin signaling pathway, such as pAKT/AKT and lipogenesis factor ACC (Figure 5f). To assess whether ROCK1 is involved in miR-324-5p-regulated lipid and glucose metabolism, ROCK1-targeted siRNA was transfected into HepG2 cells. Compared to the negative control group, ROCK1 mRNA levels were significantly reduced (Figure 5g), and Western blot analysis showed that ROCK1 knockdown inhibited the AKT/GSK signaling pathway while enhancing ACC and PEPCK expression (Figure 5h). These results are consistent with the effects observed for miR-324-5p, indicating that miR-324-5p likely plays a crucial role in improving insulin resistance through the modulation of ROCK1 and key insulin signaling nodes.

### 3.6. Adipose Tissue as a Major Source of Exercise-Induced Exosomal miR-324-5p

**Figure 6:**
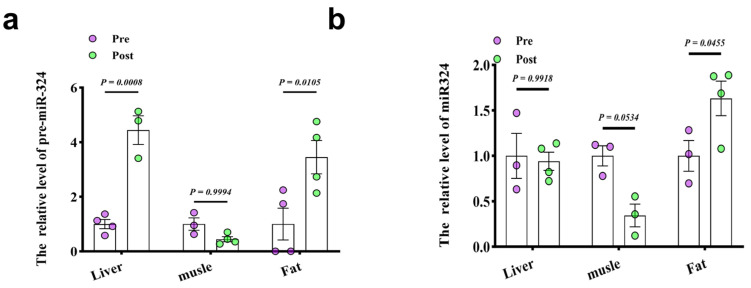
Adipose tissue as the primary source of exercise-induced exosomal miR-324-5p. (**a**,**b**) Relative levels of pre- and mature miR-324 in liver, muscle, and adipose tissue, with significant post-exercise increases in adipose tissue, suggesting it as the primary source.

To further investigate the source of circulating exosomal miR-324-5p induced by long-term exercise, we examined three major insulin-sensitive organs: liver, muscle, and adipose tissue. Our findings revealed that prolonged exercise significantly altered mature and precursor miR-324-5p levels in all three tissues, with the most notable changes observed in adipose tissue (Figure 6a,b). Although pre-miR-324 increased in the liver, mature miR-324 levels remained stable, suggesting that the liver might primarily act as a recipient of exosomal miR-324 rather than producing it endogenously in response to exercise. Meanwhile, the modest and nonsignificant decrease in muscle miR-324 expression indicates that miR-324 may not play a central role in skeletal muscle adaptation to aerobic training, instead highlighting a targeted role in adipose–liver communication. Collectively, these results point to a sophisticated, tissue-specific regulatory network in which miR-324 serves as a metabolic mediator, influencing liver function primarily through an adipose-derived exosomal pathway.

### 3.7. Elevated Exosomal miR-324 Levels in Athletes Compared to Sedentary Controls

**Figure 7:**
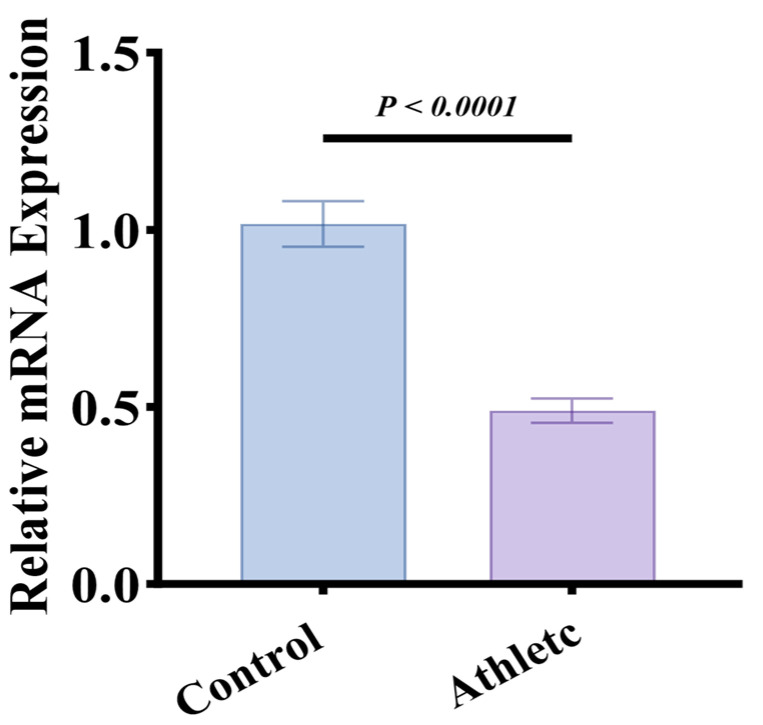
Elevated exosomal miR-324-5p levels in elite athletes compared to sedentary controls. Plasma levels of exosomal miR-324-5p in athletes vs. sedentary controls, showing significantly higher levels in athletes, indicating that sustained physical training may upregulate miR-324-5p expression.

We further examined exosomal miR-324-5p levels across different populations. Results showed that elite athletes with long-term exercise training had significantly higher exosomal miR-324-5p levels compared to sedentary controls, indicating that sustained physical training may modulate miR-324-5p expression levels, potentially impacting metabolic and cognitive responses associated with exercise adaptation (Figure 7).

## 4. Discussion

This study yielded two major findings. First, circulating exosomes derived from long-term exercise convey protective signals that significantly ameliorate insulin resistance (IR). Second, we identified adipose tissue-derived exosomal miR-324-5p as a key molecule that significantly improves hepatic metabolism by modulating ROCK1-related glucose and lipid metabolic signaling pathways and enhancing p-Akt-related survival signaling.

Long-term exercise enhances fatty acid oxidation capacity and mitochondrial function. Studies in humans have demonstrated increased hepatic and muscular fat oxidation capacity following long-term aerobic exercise [27,28,29]. Similarly, animal models have shown enhanced mitochondrial biogenesis and oxidative capacity under comparable exercise regimens [30,31]. In our study, we found that long-term exercise significantly increased the capacity for oxidizing fat in the liver and muscle, which is closely associated with greater mitochondrial oxidative capacity and biogenesis [32,33]. Exercise-induced mitochondrial biogenesis enhances cellular energy metabolism efficiency, promotes lipid oxidative utilization, and reduces fat accumulation in the liver and muscle, thereby improving insulin sensitivity [34].

Exercise-induced myokines and systemic metabolic regulation. During exercise, skeletal muscle secretes a series of cytokines known as myokines, including IL-6, IL-15, apelin, musclin, and BDNF [35,36]. These myokines, collectively termed “exerkines”, act in autocrine, paracrine, and endocrine manners to form a complex inter-organ network that contributes to systemic metabolic health. The study by Laurens et al. [37]. highlighted the crucial role of these exercise-released myokines in the control of energy metabolism, supporting our view that exercise improves metabolic function through multiple mechanisms.

Adipose tissue plays a critical role in energy regulation. Our study found that exercise significantly increased the levels of adipose tissue-derived exosomal miR-324-5p and observed elevated precursor miR-324-5p levels in adipose tissue, suggesting that adipose tissue is the primary source of miR-324-5p. The stable levels of miR-324-5p in the liver indicate that the liver is more likely a target organ of exosomal miR-324-5p rather than a tissue producing endogenous miR-324-5p post-exercise. This exosome-mediated transport process promotes the maintenance of metabolic homeostasis by modulating hepatic lipid metabolism or insulin sensitivity.

The strength of this study lies in revealing a new mechanism by which long-term exercise improves IR and regulates hepatic metabolism through adipose tissue-derived exosomal miR-324-5p. This fills a gap in previous research regarding long-term exercise and exosome-mediated inter-organ signal transmission. Compared with prior studies that mainly focused on short-term exercise effects, our research emphasizes the importance of long-term regular aerobic exercise in metabolic health and provides new ideas for developing exosome miRNA-based interventions for metabolic diseases.

However, this study also has some limitations. We lacked animal models or in vivo pharmacological inhibitors that can specifically suppress exosome production, making it impossible to further explore the protective effects of specific exosomal miRNAs on IR through loss-of-function experiments. Additionally, we did not control for other influencing factors such as diet in the 12-week moderate-to-high-intensity exercise model, which is suitable for middle-aged and elderly individuals.

The high-fat diet (HFD) mouse model offers high accuracy in studying early to moderate stages of MAFLD, especially in replicating steatosis, insulin resistance, and hepatic inflammation [38,39]. However, its accuracy decreases when attempting to reproduce advanced cirrhosis and fibrosis [40]. Therefore, future studies should consider using more precise models and exploring whether other types of exercise provide metabolic protection through similar exosomal mechanisms.

## 5. Conclusions

This study demonstrates that a 12-week aerobic Baduanjin training program significantly reduces liver fat content and improves insulin sensitivity in patients with metabolic-associated fatty liver disease (MAFLD) and in obese mice fed a high-fat diet. We identified that exercise-induced exosomal miR-324, derived from adipose tissue, regulates glucose and lipid metabolism by targeting ROCK1, unveiling a novel mechanism by which exercise improves metabolic health through exosomal miRNAs. Our findings indicate that adipose tissue is the primary source of miR-324, while the liver acts as its target organ, facilitating systemic metabolic homeostasis via exosome-mediated signaling. This study not only highlights an innovative approach combining traditional Baduanjin with modern aerobic exercise but also provides a theoretical basis for developing exosome-based therapeutic strategies for metabolic diseases.

## Figures and Tables

**Table 1:** Effects of 12-week aerobic exercise on various metabolic parameters.

	12-Week Aerobic Exercise			
	Baseline	EChowpoint	Change	*p*
Age	60.47 ± 3.76			
Height	1.6 ± 0.03			
Weight	68.55 ± 13.18	65.01 ± 11.84	–3.54	0.2675
BMI	26.69 ± 4.50	25.31 ± 4.02	–1.34	0.2161
SBP	133.47 ± 12.92	124.07 ± 11.78	–9.4	0.06
DBP	85 ± 8.57	79.87 ± 8.23	–5.13	0.139
Total serum protein	74.25 ± 5.07	74.4 ± 3.59	0.15	0.7086
TRIG	1.81 ± 0.73	1.46 ± 0.57	–0.35	0.2733
TCHOL	4.92 ± 0.89	4.43 ± 0.80	–0.49	0.2112
LDL-CH	2.80 ± 0.81	2.52 ± 0.73	–0.28	0.4405
HDL-CH	1.28 ± 0.23	1.40 ± 0.26	0.12	0.2744
GLU	5.58 ± 0.98	5.23 ± 0.75	–0.35	0.3083
ApoB	0.99 ± 0.20	0.89 ± 0.18	–0.1	0.305
AST	24.4 ± 9.11	22.2 ± 8.55	–2.2	0.686
ALT	24.67 ± 13.57	22.4 ± 12.81	–2.27	0.7739
Fasting insulin	20.8 ± 2.93	17.53 ± 2.23	–3.27	0.001
HOMA-IR	5.21 ± 1.37	4.11 ± 0.95	–1.1	0.0145
QUICKI	0.28 ± 0.01	0.29 ± 0.01	0.01	0.0136
HbA1c	6.61 ± 0.42	6.18 ± 0.31	–0.43	0.0038
Fasting C-peptide	3.06 ± 0.46	2.57 ± 0.35	–0.49	0.0024
VO_2_ max	22.7 ± 1.96	26.03 ± 2.47	3.33	0.0002
Visceral fat percentage	37.83 ± 1.74	32.73 ± 1.88	–5.1	
*OGTT parameters*				
Glucose AUC (mmol/L × 120 min)	1228.53 ± 126.55	1070.38 ± 263.31	–158.15	0.0004

Key parameters measured before and after 12 weeks of aerobic exercise training include weight, BMI, blood pressure (SBP, DBP), lipid profile (TRIG, TCHOL, LDL-CH, HDL-CH), fasting glucose (GLU), fasting insulin, HOMA-IR, QUICKI, HbA1c, fasting C-peptide, VO_2_ max, and visceral fat percentage. Significant improvements were observed in fasting insulin, HOMA-IR, QUICKI, HbA1c, fasting C-peptide, VO_2_ max, and glucose AUC, highlighting the potential of aerobic exercise in improving insulin sensitivity and reducing liver fat in MAFLD patients. Data are presented as mean ± SD. SBP refers to systolic blood pressure, and DBP refers to diastolic blood pressure. TRIG represents triglycerides, while TCHOL indicates total cholesterol. LDL-CH and HDL-CH stand for low-density lipoprotein cholesterol and high-density lipoprotein cholesterol, respectively. GLU denotes fasting glucose, ApoB refers to apolipoprotein B, and AST and ALT indicate aspartate aminotransferase and alanine aminotransferase, respectively. HOMA-IR represents the homeostatic model assessment of insulin resistance, while QUICKI is the quantitative insulin sensitivity check index. HbA1c refers to hemoglobin A1c, VO_2_ max is maximum oxygen consumption, OGTT stands for oral glucose tolerance test, and AUC indicates the area under the curve.

## Data Availability

The original contributions presented in this study are included in the article/Supplementary Material. Further inquiries can be directed to the corresponding author.

## Supplementary Materials

The following supporting information can be downloaded at: https://www.mdpi.com/article/10.3390/metabo14120692/s1, Figure S1: Effects of Exercise on Tissue Weights, Serum Biochemistry, and Protein Expression in Mice Fed a High-Fat Diet.
