# Supplementary figures and images for: Long-Term Aerobic Exercise Enhances Hepatoprotection in MAFLD by Modulating Exosomal miR-324 via ROCK1
Source: Metabolites. 2024 Dec 9;14(12):692. doi: 10.3390/metabo14120692 (PMC11679935; doi:10.3390/metabo14120692)

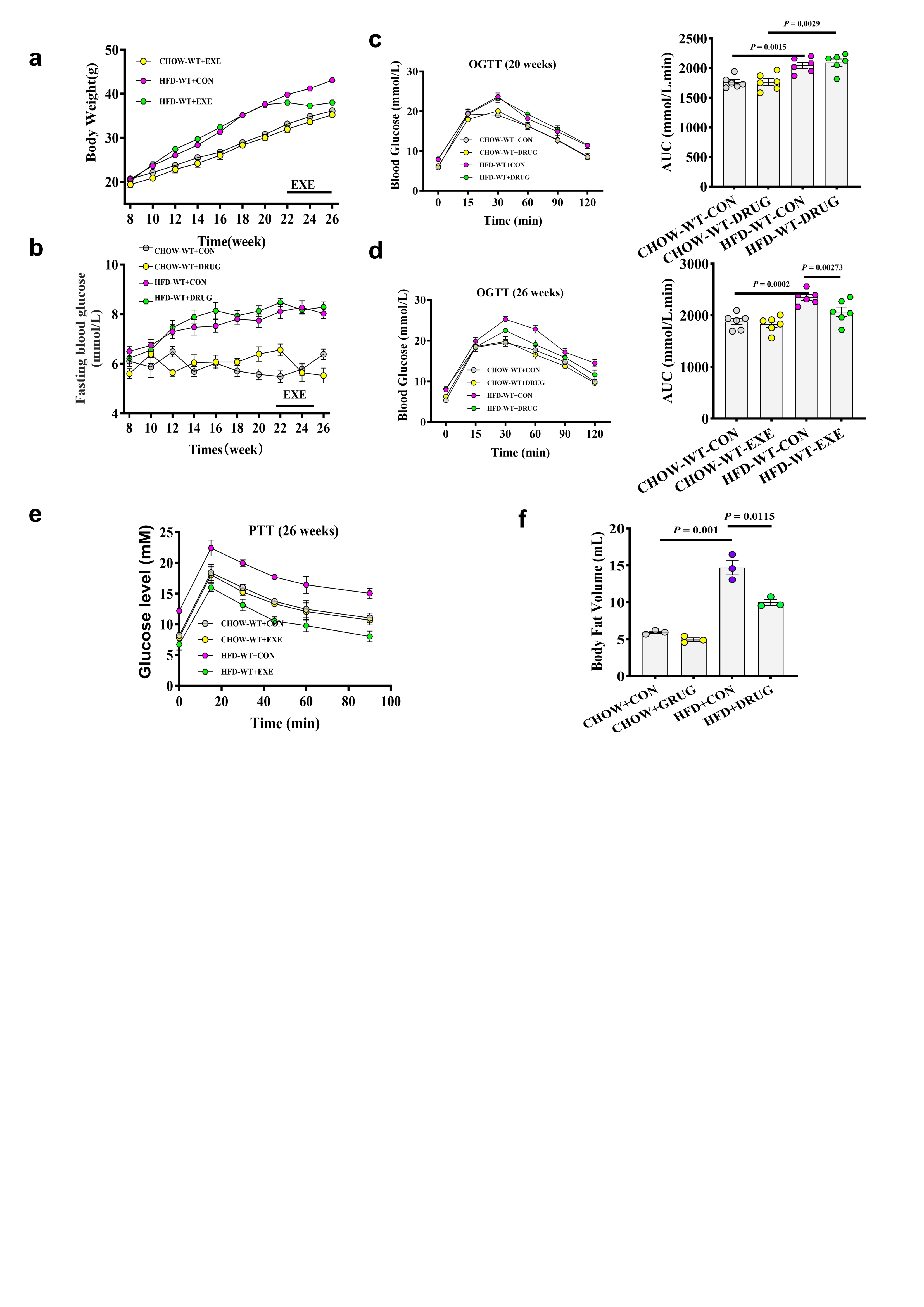

Supplement: Supplementary file 1 [file metabolites-14-00692-s001.zip › metabolites-3331580-Figure S1.tif]
